# Distracted Driving, A Major Preventable Cause of Motor Vehicle Collisions: “Just Hang Up and Drive”

**DOI:** 10.5811/westjem.2015.10.28040

**Published:** 2015-12-11

**Authors:** Christopher A. Kahn, Victor Cisneros, Shahram Lotfipour, Ghasem Imani, Bharath Chakravarthy

**Affiliations:** * University of California, San Diego, Department of Emergency Medicine, San Diego, California; † University of California, Irvine, Department of Emergency Medicine, Orange, California

## Abstract

For years, public health experts have been concerned about the effect of cell phone use on motor vehicle collisions, part of a phenomenon known as “distracted driving.” The Morbidity and Mortality Weekly Report (MMWR) article “Mobile Device Use While Driving - United States and Seven European Countries 2011” highlights the international nature of these concerns. Recent (2011) estimates from the National Highway Traffic Safety Administration are that 10% of fatal crashes and 17% of injury crashes were reported as distraction-affected. Of 3,331 people killed in 2011 on roadways in the U.S. as a result of driver distraction, 385 died in a crash where at least one driver was using a cell phone. For drivers 15–19 years old involved in a fatal crash, 21% of the distracted drivers were distracted by the use of cell phones. Efforts to reduce cell phone use while driving could reduce the prevalence of automobile crashes related to distracted driving. The MMWR report shows that there is much ground to cover with distracted driving. Emergency physicians frequently see the devastating effects of distracted driving on a daily basis and should take a more active role on sharing the information with patients, administrators, legislators, friends and family.

## CDC MORBIDITY & MORTALITY WEEKLY REPORT FINDINGS

In the March 15, 2013, issue of the *Morbidity and Mortality Weekly Report* (MMWR), the Centers for Disease Control and Prevention (CDC) reported information on self-reported mobile device use while driving in the United States and seven European countries.^1^ The report described that among drivers ages 18–64, the prevalence of talking on a cell phone while driving at least once in the past 30 days ranged from 21% in the United Kingdom (UK) to 69% in the U.S. In addition, the prevalence of drivers who read or sent text or e-mail messages while driving at least once in the past 30 days ranged from 15% in Spain to 31% in both Portugal and the U.S.

To describe these trends, the CDC analyzed data from the 2011 EuroPNStyles and HealthStyles surveys, which were created by a worldwide social marketing and public relations firm.^1^ These surveys were conducted among adults (age >18 years) to examine health-related attitudes and behaviors. In the U.S., the HealthStyles survey used was from September 30 to October 5, 2011. They randomly sampled 5,315 people, with 70% (3,696) completing the HealthStyles survey. The HealthStyles survey data were weighed to match nine characteristics (sex, age, annual household income, race/ethnicity, household size, education, U.S. census region, metropolitan status, and prior internet access) of the U.S. current population. The EuroPNStyles survey was conducted in July 2011 in Belgium, France, Germany, the Netherlands, Portugal, Spain, and the UK. Samples were selected to match each country’s census proportion for age and sex. All countries reached 1,700 adults, with the exception of Spain and Portugal, which were only able to attain 850 adults. Both surveys asked if participants had driven in the past 30 days. If so, then they would continue to ask, “In the past 30 days, how often have you talked on your cell phone while you were driving?” and “In the past 30 days, how often have you read or sent a text message or e-mail while you were driving?” Survey participants had the following response choices: “never,” “just once,” “rarely,” “fairly often,” and “regularly.”

The U.S. results showed that, in 2011, more than two-thirds (68.7%) of drivers between ages 18–64 years had talked on their cell phone while driving at least once in the past 30 days. European percentages ranged from 20.5% in the UK to 59.4% in Portugal. Furthermore, 31.2% of U.S. drivers in the same age range reported they had read or sent text or e-mail messages while driving at least once in the past 30 days versus Europe’s percentages ranged from 15.1% in Spain to 31.3% in Portugal. Finally, few differences by sex were observed in the U.S., although there was a significant difference by age. A larger percentage of people aged 25–44 years reported talking on a cell phone, reading or sending texts or email messages while driving compared to those aged 55–64 years.

The editorial note portion of the report listed seven limitations. First, both surveys might not be representative of each of the eight countries due to the sampling approaches not being completely random. Second, the HealthStyles sample was not dependent on computer and internet access, which was not the case for the EuroPNStyles sample; this could affect representation in each country. Third, findings could be subject to non-response bias. Fourth, findings might be subject to social desirability bias, due to different laws in each country that could influence use of devices while driving. Fifth, surveys did not ask about cell phone ownership or capabilities, so the “never” response could include people that do not have a cell phone. Sixth, recall bias has to be taken into consideration, because estimates were reported on driving in the past 30 days. Finally, the study was restricted to a certain age population (ages 18–64 years), which does not represent the whole driving population in each of the countries.

## COMMENTARY

“This is the story of how my daughter Liz’s car accident from texting while driving changed our lives forever. If you get a text, don’t look at it while you’re driving. It’s not worth it,” is the caption under the video posted to the National Highway Traffic Safety Administration (NHTSA)YouTube page.^2^ The video tells the story of Liz Marks, a young woman in high school whose life was dramatically changed after she was involved in a crash caused by reading a text while driving. The crash left her blind in one eye, hard of hearing, with a diminished sense of smell and no longer able to create tears or fall asleep without medication. Liz shares her story in the video in hopes of reminding people what is at stake when someone texts while driving.

For years, public health experts have been concerned about the effect of cell phone use on motor vehicle crashes. While the specifics have changed over time [What about handheld vs. hands-free? What about texting? What about younger vs. older drivers?] what has not changed is the concern that people focusing on their phone are not focusing on their driving. The recent MMWR article, “Mobile Device Use While Driving – United States and Seven European Countries, 2011”– helps highlight the international nature of these concerns.^1^

Texting and making/taking calls on a cell phone are part of the general category of risky driving behavior referred to as “distracted driving.” Recent estimates from the NHTSA from 2011 are that 10% of fatal crashes and 17% of injury crashes were reported as distraction-affected.^3^ Of 3,331 people killed that year on public roadways in the U.S. as a result of driver distraction, 385 died in a crash where at least one driver was using a cell phone (12% of all distraction-affected fatal crashes). For drivers 15–19 years old involved in fatal crashes, 21% of the distracted drivers were distracted by the use of cell phones.

The HealthStyles and EuroPNStyles surveys reported by the MMWR demonstrate that anywhere from 20–70% of adults 18–64 years of age used a cell phone at least once in the 30 days prior to the survey. Text messaging use was closer to 15–30% across the eight countries involved. Although there are clearly limitations to these data, such as social desirability bias and recall bias, these rates appear to be reasonable approximations of actual use based on smaller samples subjected to actual observation in other studies.

While there are limited resources for injury prevention efforts, this is a worthwhile topic on which to focus those resources. For every traumatic death, there is a much larger number of injuries. For example, although “only” 3,331 people died in 2011 as a result of distraction-affected crashes, another 387,000 (estimated) were injured.^3^ When we start to consider the societal impact of hundreds of thousands of injured people, time lost from work, time and expense required to be evaluated and treated by medical professionals, to repair vehicles, and factors harder to quantify, such as loss of ability to participate in various recreations and family activities, these all add up to a significant problem.

Younger drivers, who are at higher risk for these behaviors, generally underestimate the risk of distracted driving. A 2013 study noted that just under half of surveyed U.S. high school students 16 or older reported texting while driving at least once in the prior 30 days.^4^ These students were also more likely to not always wear their seatbelts, ride with a driver who had been drinking, and to drive after drinking alcohol. In fact, the more often a student texted, the more likely he or she would engage in other risky behaviors. The prevalence of driving after drinking alcohol was 3% in the group that did not text even once in the prior 30 days while driving. However, this rate was 34% in the group that texted while driving on every one of the prior 30 days.

We know that younger drivers are overrepresented in fatal crashes. In 2007, while they comprised 9% of the U.S. population and 6% of the licensed drivers, 19% of all crash fatalities in the U.S. were related to young-driver crashes.^5^ We know that younger drivers have more difficulty dealing with roadway hazards and perceiving traffic threats.^6^ In response, many states are implementing legislation to restrict the driving privileges of younger drivers through measures such as graduated drivers licenses and special restrictions on cell phone use by novice drivers. As of mid-2015, 38 states and the District of Columbia (DC) banned all cell phone use by novice drivers. 46 states (as well as DC, Puerto Rico, Guam, and the U.S. Virgin Islands) ban texting for all drivers, with another two having a texting ban that applies only to novice drivers. The states that allow teen texting are Arizona and Montana.^7^ Graduated drivers licenses restrict carrying passengers and driving at night for the period between learning and full-privilege stage. This is in effect in some form in all 50 states and DC.^8^

Older, more experienced drivers do not necessarily have a better grasp of the true level of impairment that texting and talking on the phone involve. One study published in 2008 showed that the drivers who felt they were least affected by distractions turned out to be the most distracted as measured by velocity control and traffic signal reaction tasks.^9^ A later survey of a convenience sample of 1,857 adults showed that 63% of respondents felt they could drive safely while distracted.^10^

How is it that despite years of advocacy, legislation, and exposure to news reports, so many people still do not seem to “get it”? The evidence is overwhelming that distracted driving is dangerous. It leads to decrements in traffic flow and overall safety (especially when texting).^11^ Texting negatively affects specific driving tasks such as lane management and velocity control.^12^ Handheld cell phones physically restrict head movement and range of gaze, and cell phone conversations have been shown to artificially constrict peripheral awareness as measured by visual fields.^13^,^14^ Despite several states in which legislators apparently feel otherwise, driving while using a hands-free device is not actually safer than using a handheld phone.^15^ Simple phone conversations worsened braking time and velocity control similarly to those with a blood alcohol concentration (BAC) of 0.04 in a crossover study of a dozen Australian volunteers. Furthermore, those who texted and engaged in more cognitively demanding conversations were more similar to those with BACs of 0.07 and 0.10.^16^ For comparison’s sake, even though a BAC of 0.04 is legal throughout the U.S., the risk of fatal motor vehicle injury increases in a steady dose-response relationship. With each and every drink including the first, the odds ratio (OR) for fatal injury is 1.74 for every 0.02 increase in BAC, which is roughly one drink. However, at BAC level of 0.08, the OR was 13.0.^17^ Even something as simple as hearing a cell phone’s ringtone causes a slower reaction time.^18^

Evidence of these dangers has been widely available for years. As suggested by Atchley et al, the problem may be that social norms have not yet changed to make distracted driving unappealing.^19^ As a comparison, it is widely accepted now that drinking and driving is not a good idea. However, despite reports from the turn of the century that alcohol and automobiles did not mix well, it was not until the 1980s with the founding of Mothers Against Drunk Driving, related media, and governmental campaigns that the public started paying attention. From 1980 to 1985, over 700 new drunk-driving laws hit the books across the U.S., and drunk-driving deaths began to significantly decrease.^20^ It finally became “uncool” to drink and drive. Similar efforts toward distracted driving will likely be a critical aspect of changing the perceptions of modern drivers. William Haddon, Jr., the first administrator of what became the NHTSA, is famous in injury prevention studies. His matrix consists of personal attributes (human factors), vector attributes (equipment), and environmental attributes (social norms and legislation) examined in pre-, intra-, and post-injury phases.^21^ Considering distracted driving in this framework, changing attitudes can be just as important as changing the technology in the car.

The MMWR report on the HealthStyles and EuroPNStyles surveys shows that we have a lot of ground to cover. Fortunately, the evidence is on our side. We should certainly be sharing that evidence – gently – with those we meet in our emergency departments who have arrived after a motor vehicle collision. However, we should also be sharing it with our administrators, our legislators, and our friends and families. Emergency physicians see the devastating effects of distracted driving frequently, and should be a part of the solution rather than just noting the problem. We also need to ensure that we do not simply ignore it when it comes to our own driving behaviors. As difficult as it is, we all need to just hang up and drive.
